# Molecular Imaging for Personalized Medicine

**DOI:** 10.1155/2016/5170159

**Published:** 2016-08-29

**Authors:** James Russell, Jie Tian, Seigo Kinuya, Baozhong Shen, Xiao-Feng Li

**Affiliations:** ^1^Memorial Sloan Kettering Cancer Center, New York, NY, USA; ^2^Chinese Academy of Sciences, Beijing, China; ^3^Kanazawa University, Kanazawa, Japan; ^4^Harbin Medical University, Harbin, China; ^5^University of Louisville, Louisville, KY, USA

This issue is devoted to molecular imaging in the context of personalized medicine. Recent advances in biology, particularly genomic and expression screens, have made it possible to hope that treatment can be matched very specifically to the patient's disease, as characterized at the molecular level. In oncology, where hopes for personalized medicine are perhaps highest, tumors will be treated based on the specific mutations that drive their unrestrained growth, rather than on their site of origin. For all branches of medicine, there is also the potential for matching drug dosage to the individual's drug metabolism profile, established through genetic analysis.

Along with genomic information, molecular imaging will be crucial in the development of personalized medicine. Molecular imaging—essentially any imaging procedure that delivers some information on a disease state, beyond the purely anatomical—is an incredibly diverse area of research, covering every disease state and imaging modality, with astonishing innovation in developing novel imaging agents. However, there are three aims that commonly emerge in the literature: to image some physiological/biological state that will be diagnostic/predictive; to observe treatment response; and to image the accumulation of drug in the lesion, an approach known as theranostics.

Appropriately, all these aims are represented in the six articles featured here. Dr. X. Li et al. (Shenyang, China) report the successful production of a ^68^Ga labeled rhodamine based probe for PET imaging of mitochondrial potential in tumors, with the biology of probe uptake still to be clarified. Dr. W. Zhou et al. (Hohhot, China) demonstrate that N-11C methyl dopamine is a promising PET imaging agent for cardiac reperfusion injury, comparing it with a commonly used PET tracer of perfusion, ^13^N ammonium in a swine model. X. Wang et al., also from Hohhot, present a comparison between ^99m^Tc labeled Annexin-V and methyl diphosphonate, in the detection of femoral head osteonecrosis in glucocorticoid treated rabbits, which indicates an advantage for Annexin-V in earlier detection of pathologic changes. B.-C. Ahn (Daegu, Republic of Korea) presents a review of theranostic imaging in the context of radioiodine treatment of differentiated thyroid cancer, while G. Liu et al. (Shenyang) make a practical contribution to this disease by demonstrating the superiority of ^131^I imaging compared to ^99m^TcO_4_^−^ in identifying remaining tissue in patients who have undergone surgery. Finally, Q. Liu et al. (Shanghai) publish a meta-analysis of ^18^F-FDG PET/CT imaging and MRI in determining pathological complete response in breast cancer patients receiving neoadjuvant chemotherapy.

We hope that the reader will find something stimulating in this selection from an exciting and rapidly expanding area of research.

